# Incidental Finding of Gastrointestinal Stromal Tumors in the Appendix

**DOI:** 10.7759/cureus.83413

**Published:** 2025-05-03

**Authors:** Safa Alshaikh, Aalaa Mubarak, Noor Alosaif

**Affiliations:** 1 Department of Pathology, Salmaniya Medical Complex, Manama, BHR; 2 Department of Pediatric Surgery, Maternity and Children Hospital, Dammam, SAU

**Keywords:** appendiceal neoplasm, appendicectomy, appendix, gastrointestinal stromal tumor, gist

## Abstract

A 37-year-old man who presented with upper abdominal pain was diagnosed with acute appendicitis. He underwent an open appendicectomy. The intraoperative findings included only an acutely inflamed appendix. Histopathological examination of the appendix showed an incidental finding of gastrointestinal stromal tumors (GISTs). This paper highlights the contributory factors that are assessed in the risk assessment of appendiceal GISTs. However, as these entities are rare, an accurate risk stratification and progression assessment tool has not been established yet.

## Introduction

Gastrointestinal stromal tumors (GISTs) are rare entities that arise from the interstitial cells of Cajal [1,2]. They account for less than 1% of gastrointestinal tumors [3] and have a median age of 65 years [2].

The stomach and the small intestine are the most commonly reported sites for GISTs [3]. However, it can also occur in other areas of the gastrointestinal tract. Primary appendiceal GISTs are misdiagnosed as acute appendicitis and treated with appendectomy, as they can present with appendicitis-like symptoms [4]. Furthermore, it can be an incidental finding on imaging, during operation for other diseases, or at autopsy [5].

GISTs are reported to behave in different patterns regarding the location, despite the similar histopathologic and molecular features [6], and can show variable malignant potential. Stomach GISTs that are incidentally found under the microscope rarely progress. However, mitotically active and large GISTs show a high risk for progression, especially if they occur in the small bowel [7]. Indeed, risk stratification and prognostication for the appendicular GISTs are still unclear and are derived from data from more common sites [6].

## Case presentation

Case history

A 37-year-old man presented to Salmaniya Medical Complex, Manama, Bahrain, with a one-day history of abdominal pain, which was associated with vomiting. The patient had no significant medical or surgical history. Upon admission, the patient was vitally stable, with a heart rate of 88 beats per minute, a blood pressure of 120/75 mmHg, and a temperature of 37.2°C.

On physical examination, the patient exhibited right iliac fossa tenderness with positive rebound tenderness, a key finding suggestive of peritoneal irritation typically seen in acute appendicitis. The tenderness is localized to the McBurney’s point, which is classically associated with appendicitis. There were no signs of peritonitis elsewhere in the abdomen, and no palpable masses were noted on abdominal examination.

Management

Laboratory results showed a slight elevation in the white blood cell count at 13 × 10^3^/µL, which is consistent with the inflammatory response seen in acute appendicitis. Other lab values, including electrolytes, liver function tests, and renal function, were unremarkable.

Alvarado score

To assist in the diagnosis of acute appendicitis, the Alvarado score was calculated based on the patient's clinical findings. With a total score of 9 points, the patient was classified as having a high probability of acute appendicitis. The score was determined from the following: right iliac fossa tenderness (2 points), rebound tenderness (1 point), nausea/vomiting (1 point), anorexia (1 point), and leukocytosis (2 points). A score of 7 or more strongly suggests the need for surgical intervention, and given this patient’s score of 9, it supported the decision to proceed with an open appendicectomy.

An abdominal CT scan was ordered to rule out other potential causes of abdominal pain, but unfortunately, there was no formal report generated by the system. Based on the clinical examination and laboratory findings, the decision was made to proceed with surgery. The patient underwent an open appendicectomy. Intraoperative findings revealed an appendix that was acutely inflamed, with no signs of perforation, abscess formation, or gangrenous changes. These findings suggest that the patient was in the early stages of appendicitis, where the appendix had not yet perforated or developed significant complications.

Histopathology assessment

The appendix was sent for histopathological examination, revealing gross findings that included a congested serosa and a well-circumscribed, firm nodular area, which was 1 cm away from the base resection margin. The background appendix showed features of acute appendicitis, which were characterized by the following: 1) neutrophilic infiltration of the mucosa, submucosa, and muscularis propria, indicative of the acute inflammatory process, and 2) edematous changes and vascular congestion of the appendix wall, which are common early histopathological findings in appendicitis.

**Figure 1:**
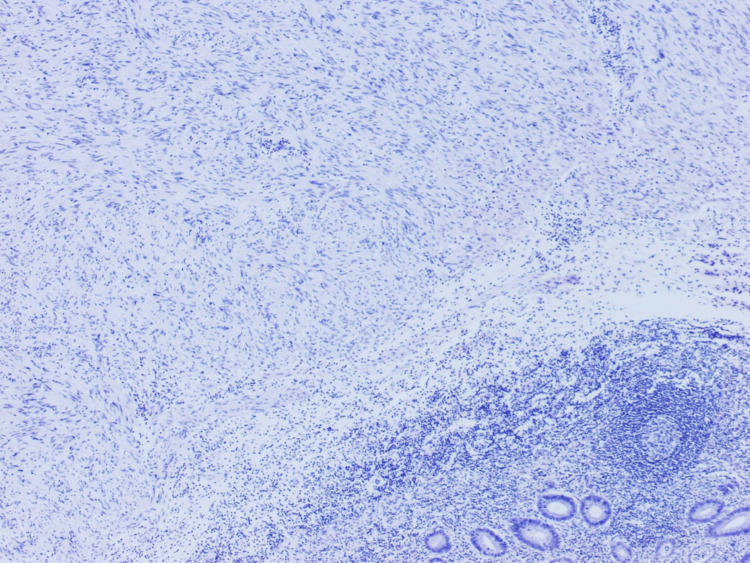
An H&E-stained slide, examined under a magnification of ×4 H&E: hematoxylin and eosin

**Figure 2:**
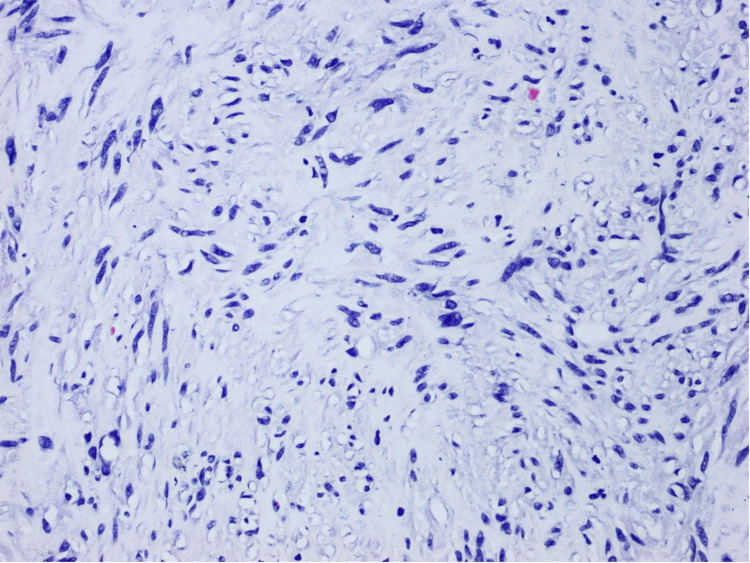
An H&E-stained slide, examined under a magnification of ×20 H&E: hematoxylin and eosin

**Figure 3:**
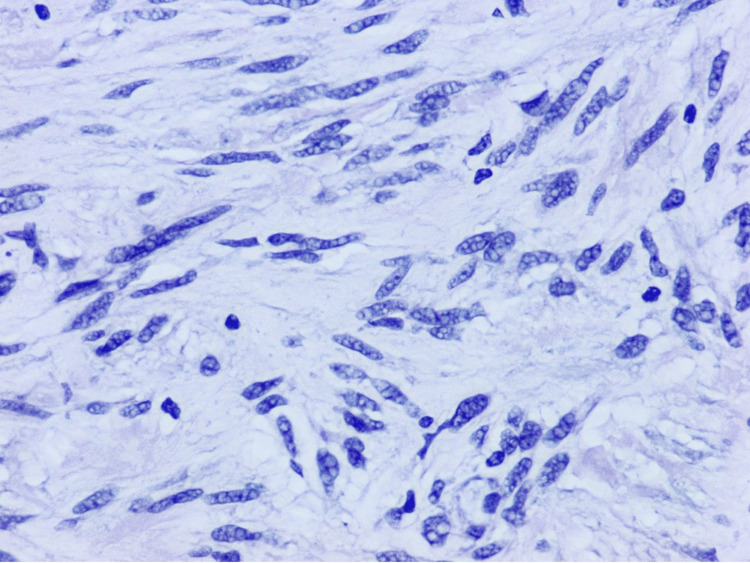
An H&E-stained slide, examined under a magnification of ×40 H&E: hematoxylin and eosin

The tumor was identified in a separate, well-defined nodule composed of proliferative, eosinophilic spindle cells with skeinoid fibers (Figures 1-3), a finding unrelated to the acute appendicitis but of interest due to its rarity in appendiceal pathology.

**Figure 4:**
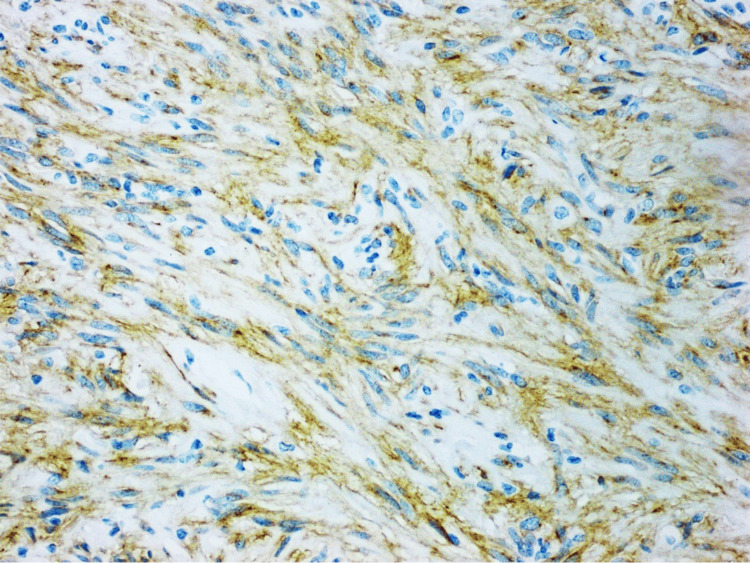
KIT (CD117) showing strong cytoplasmic staining of the lesional cells, examined under a magnification of ×20

**Figure 5:**
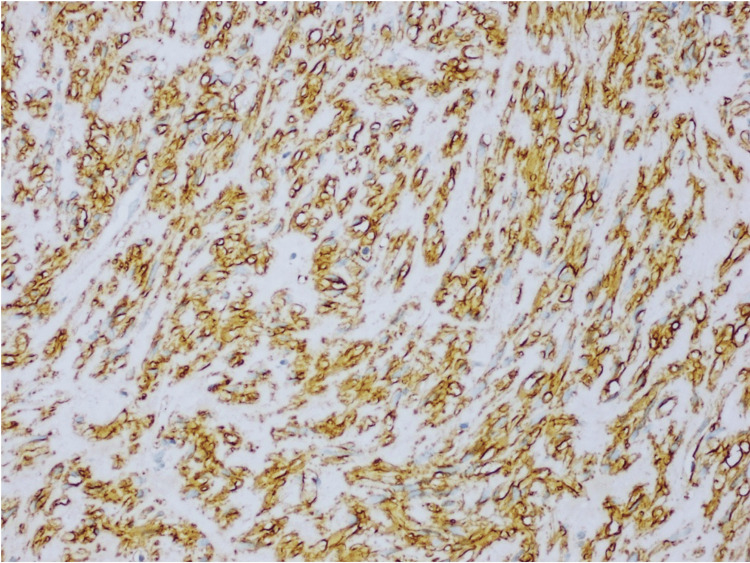
CD34 showing strong cytoplasmic staining of the lesional cells, examined under a magnification of ×20

**Figure 6:**
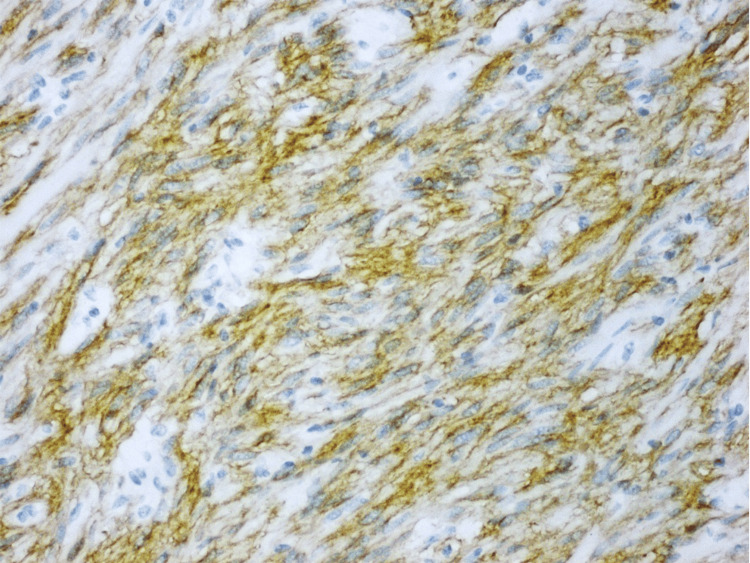
DOG1 showing strong cytoplasmic staining of the lesional cells, examined under a magnification of ×20

KIT (CD117), CD34, and DOG1 (ANO1) were positive in the tumor, as shown in Figures 4-6, respectively, confirming the diagnosis of GIST.

**Figure 7:**
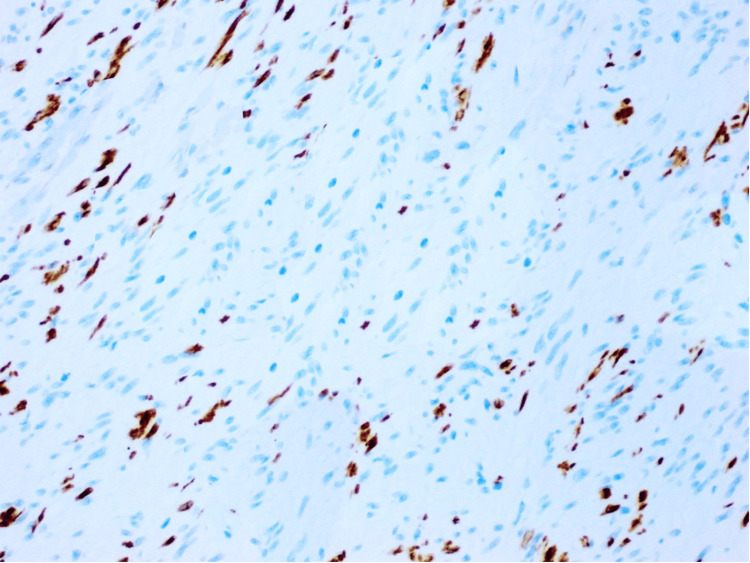
S100 showing negative staining of the lesional cells, examined under a magnification of ×20

**Figure 8:**
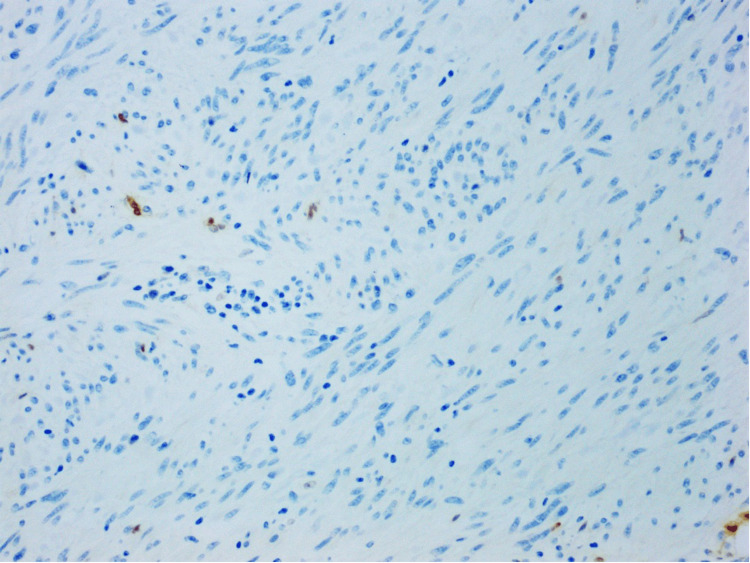
Desmin showing negative staining of the lesional cells, examined under a magnification of ×20

**Figure 9:**
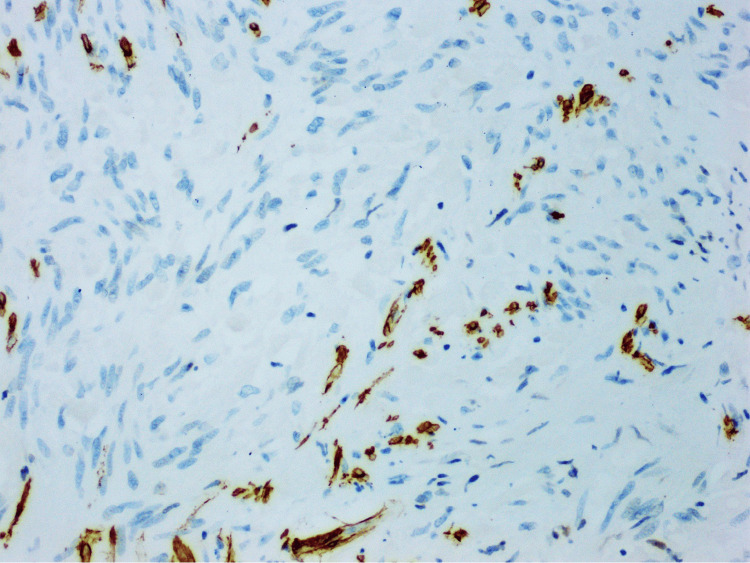
SMA showing negative staining of the lesional cells, examined under a magnification of ×20 SMA: smooth muscle actin

On the other hand, S100, Desmin, and smooth muscle actin were negative, as shown in Figures 7-9, respectively, which excludes other differential diagnoses, such as schwannoma and leiomyoma.

Follow-up

The patient was discharged from the surgical ward in a stable condition with a plan for routine follow-up; however, he failed to attend subsequent outpatient visits. The lack of follow-up highlights a gap in the patient’s care after surgery, which is crucial for monitoring potential long-term outcomes, particularly in cases involving unusual histopathological findings.

## Discussion

GISTs are rare neoplasms arising from the interstitial cells of Cajal, with varying potential for malignancy. These tumors are most commonly found in the stomach, small bowel, and rectum, with known risk stratification models to assess prognosis and treatment outcomes. However, when GISTs occur in less common sites, such as the appendix, the risk stratification and clinical management remain less well-established. This case report examines an appendiceal GIST, a rare and intriguing occurrence, in the context of existing literature on appendiceal GISTs and other gastrointestinal sites.

Comparison with literature on appendiceal GISTs

A cohort study by Hu et al. used the Nashville risk score (NRS), a tool that validates tumor size and mitotic count as risk factors in GISTs, including those arising in uncommon locations such as the esophagus, colon, and appendix. Hu et al. found that NRS could predict progression-free survival for GISTs in rare sites, including the appendix [8]. In this study, the appendix (n = 27) showed a low incidence of malignant progression, consistent with our case, where the tumor was small (1.6 cm) and mitotically inactive. Therefore, our case can be classified as low risk based on these findings.

Comparison with other appendiceal GIST studies

In contrast, other studies have shown that larger and mitotically active appendiceal GISTs are more likely to progress. Hu et al. highlighted that the majority of appendiceal GISTs are small and mitotically inactive, with appendectomy being curative in most cases. However, larger tumors or those with mitotic activity may present a higher risk of progression, potentially requiring cecectomy or other forms of adjuvant therapy. This is in line with our case, where the GIST was small and showed no mitotic activity, which aligned with the literature suggesting a low risk for malignant progression and no evidence of recurrence after appendectomy [7].

Risk stratification and tumor characteristics

The appendiceal GISTs, as shown in our case, often present as small, benign-appearing tumors with limited mitotic activity, aligning with findings in several studies. Blandino and Naser also concluded that for most appendiceal GISTs, appendectomy alone is an adequate treatment. In their case series, they reported a patient who had delayed appendectomy followed by cecectomy, with no recurrence or metastasis after 6.5 years of follow-up [4]. This finding supports our approach of treating small appendiceal GISTs with surgical resection, where appendectomy suffices for tumor clearance.

However, some studies, including Hu et al., have pointed out that larger appendiceal GISTs may exhibit more aggressive behavior. For example, gender, clinical presentation, and tumor necrosis have been shown to contribute to tumor progression, especially in high-risk GISTs. This suggests that even for low-risk GISTs, close monitoring and follow-up may be necessary to ensure that tumors do not progress to more aggressive forms.

Comparison with larger cohorts

Khan et al. conducted a large study of 10,833 patients diagnosed with GISTs, identifying 13 primary appendiceal GISTs (0.1%). Surgical resection was the primary treatment modality, and the five-year overall survival for these patients was 40%, which is notably lower than that of GISTs arising from more common locations like the stomach and small bowel [9]. This statistic underlines the rarity and unpredictability of appendiceal GISTs, as survival rates for this group seem to be lower despite surgery. The lower survival rate might be due to the difficulty in diagnosing these tumors early, as appendiceal GISTs are often asymptomatic or present with vague abdominal complaints, leading to delayed diagnosis and treatment.

In our case, the appendiceal GIST was detected early and was surgically resected without complications, which contrasts with Khan’s findings, where a delayed diagnosis could contribute to worse outcomes. This highlights the importance of early detection, particularly in rare sites like the appendix, where the tumor may present with subtle symptoms, and thus, more aggressive outcomes might occur if untreated.

Tumor size and mitotic rate

It is also noteworthy that most appendiceal GISTs, including the one in this case, are small in size and exhibit low mitotic activity, which is consistent with the findings of Hu et al. They have suggested that small appendiceal GISTs are typically benign, and appendectomy remains the primary treatment with a high success rate. Conversely, large appendiceal GISTs with higher mitotic rates may have a greater tendency for progression, as seen in other case studies in the literature.

In this context, our patient’s low-risk GIST does not show signs of aggressive behavior, and the outcome after surgery is favorable, which aligns with existing findings in the literature. However, given the rarity of these tumors, it remains essential to continue monitoring even low-risk cases to ensure no progression occurs.

## Conclusions

This case of appendiceal GIST supports the general consensus in the literature that most appendiceal GISTs are benign, small, and mitotically inactive, making appendectomy an effective and curative treatment. However, it also underscores the challenges in assessing the long-term prognosis and the need for long-term follow-up, as larger, mitotically active appendiceal GISTs can exhibit more aggressive behavior. The rarity of these tumors makes it crucial to continue to expand the scientific understanding of their pathophysiology and clinical outcomes through more extensive studies.
